# Real-time PCR detection of *Coxiella burnetii* in dairy products in Kwara State, Nigeria: a public health concern

**DOI:** 10.1186/s12917-024-04449-2

**Published:** 2025-01-07

**Authors:** Nusirat Elelu, Nwachukwu Raymond Chinedu, Balkees A. Yakub-Obalowu, Ismail A. Odetokun, Ahmad I. Al-Mustapha

**Affiliations:** 1 Department of Veterinary Public Health and Preventive Medicine, Faculty of Veterinary Medicine, University of Ilorin, Ilorin, Kwara State Nigeria; 2 Department of Food Hygiene and Environmental Health, Faculty of Veterinary Medicine, University of Helsinki, Helsinki, Finland; 3 Department of Veterinary Public Health and Preventive Medicine, Faculty of Veterinary Medicine, University of Ibadan, Ibadan, Oyo State Nigeria; 4 Department of Veterinary Services, Kwara State Ministry of Agriculture and Rural Development, Ilorin, Kwara State Nigeria

**Keywords:** *C. burnetii*, Zoonoses, Foodborne pathogen, Milk

## Abstract

**Background:**

*Coxiella burnetii* is the etiological agent of Q fever in humans, a zoonosis of increasingly important public health concern. The disease results in significant economic losses to livestock farmers and its presence in ready-to-eat dairy products poses a public health threat to consumers.

**Aim:**

This study aimed to detect *Coxiella burnetii* in dairy products in Kwara State, Nigeria.

**Methods:**

A cross-sectional study was performed to estimate the apparent and true prevalence of *C. burnetii* in dairy cattle in selected local government areas (LGAs) of Kwara State, Nigeria. A total of 51 traditional Fulani transhumance farms were sampled across three regions: 27 farms in Ifelodun LGA, 9 in Ilorin East LGA, and 15 in Moro LGA. Four pooled milk samples were collected aseptically from lactating cows on each farm, totaling 204 milk samples. We screened the pathogen using real-time PCR that targeted the *IS1111* element in the 51 pooled raw milk samples from selected farms and 18 cheese samples from rural markets within the study area.

**Results:**

The overall apparent prevalence of *C. burnetii* in milk and cheese was 18.8%. Both dairy products showed similar prevalence with a relatively higher positivity rate in cheese (22.2%, *n* = 4/18) than in raw milk samples (17.6%, *n* = 9/51). There were significant differences in the burden of *C. burnetii* in milk between the three LGAs with higher prevalence in Moro LGA (*p* < 0.05).

**Conclusions:**

The presence of *C. burnetii* in dairy products poses a direct risk of milk-borne zoonotic disease transmission to humans. Public education for the stakeholders in the dairy value chain should be intensified and the public should desist from consumption of unpasteurized milk. More molecular studies are needed to further study and characterize the *C. burnetii* genotypes in Nigeria.

**Supplementary Information:**

The online version contains supplementary material available at 10.1186/s12917-024-04449-2.

## Introduction

*Coxiella burnetii*, (*C. burnetii)* the causative agent of Q fever, is a highly infectious, zoonotic, obligate intracellular bacterium. It has a wide host range, including domestic and wild mammals, birds, and arthropods which serve as major reservoirs for human infections [15, 16; 24].

Transmission to humans could occur through the inhalation of aerosolized particles from contaminated birthing materials, contact with infected urine or faeces, or through the consumption of contaminated food products such as milk [30, 34, 43]. It is primarily an occupational hazard for individuals working in close contact with livestock, but can also affect the general population, especially those who consume unpasteurized dairy products such as raw milk and cheese [16, 31, 38]. In humans, the clinical manifestations of acute Q fever range from asymptomatic or flu-like illness to severe cases of pneumonia, hepatitis, or endocarditis, with a significant risk of chronic infection in immunocompromised individuals [19, 36; 31].

The review of epidemiology and transmission dynamics of *C. burnetii* across Africa revealed that the pathogen is endemic in cattle, small ruminants, and humans across the continent, with seroprevalence ranging from 4 to 55% in cattle [38]. In Nigeria, a high prevalence of 44% was recorded in a sero-epidemiological investigation of Q fever among hospitalized patients as far back as 1990 [8]. Across Nigeria, a recent review reported a pooled prevalence of 2.5–23.5% in cattle with variations across the country [28]. In Nigeria, Q fever is one of the underreported and neglected zoonotic diseases, with limited epidemiological data available. The few studies conducted were mostly based on serological evidence of anti-C. *burnetii* antibody using the iELISA, a relatively cheaper but less sensitive diagnostic (screening) test for infectious diseases when compared with real-time polymerase chain reaction (real-time PCR) [3, 28]. However, the direct detection and molecular characterization of *C. burnetii* in animal reservoirs, particularly in milk samples, remain largely unexplored in the country.

Kwara State, located in the north-central region of Nigeria, has a significant livestock population, with cattle, sheep, and goats playing a crucial role in the agricultural economy and subsistence of rural communities. The consumption of unpasteurized milk and local cheese poses a potential risk for zoonotic transmission of disease-causing pathogens such as *C. burnetii* [11]. Previously, a study reported viable *C. burnetii* in hard cheeses that were made with unpasteurized milk [5]. We had earlier conducted a seroprevalence survey of *C. burnetii* among cows to generate baseline data on *C. burnetii* as well as evaluate the risk factors and predictors that could impact *C. burnetii* transmission among cattle, sheep, and goats in the state [16]. Therefore, this study detected the presence of *C. burnetii* in milk samples selected from farms and cheese from three popular markets in Kwara State using real-time PCR. Understanding the true prevalence and molecular epidemiology of *C. burnetii* in these animal reservoirs is crucial for developing effective control and prevention strategies, ultimately safeguarding public health in Nigeria.

## Methods

### Sample size calculation

We calculated the sample size using the Thrusfield [33] formula as shown below:

n = {1.96^2^ × P_exp_ × (1-P_exp_)}/d^2^ = 1.96^2^ × 0.156 × 0.844/0.01^2^ = 203.

Where: n: is the total number of farms to visit, P_exp_: Expected herd prevalence (P_exp_) of 15.6% obtained from a previous study on *C. burnetii* carried out in Kwara State [16], and d: Desired absolute precision (d) of 5%. Therefore, a minimum of 203 milk samples were collected across the various farms sampled in Kwara state.

### Sampling method and collection

A simple random sampling of 51 pooled milk samples (*n* = 204 milk samples) were collected from three Local Government Areas (LGAs) of Kwara state; Moro, Ifelodun, and Ilorin East between January and April 2024. The 51 farms were visited and 3–4 free milk samples from lactating animals were pooled. An additional 18 cheese samples were purchased purposefully from three major markets (six cheese samples each) situated within each of the sampled LGAs of Kwara State.

**Fig. 1:**
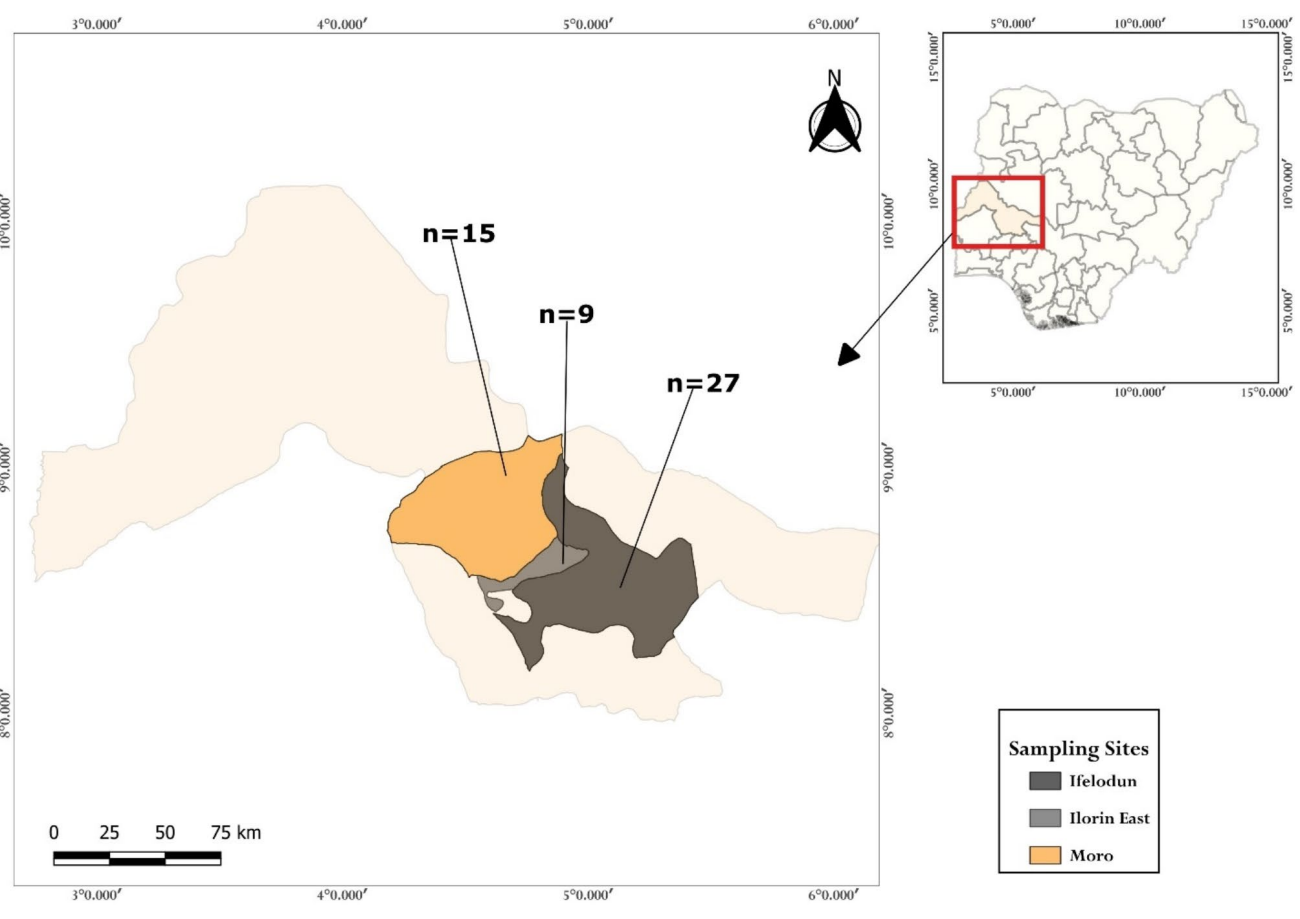
Map of Kwara State showing the sampling locations. The inset is a map of Nigeria showing Kwara State

A total of 27 cattle farms were visited in Ifelodun LGA whereas 9 and 15 farms were visited in Ilorin East and Moro LGA respectively (Fig. 1). All the farms were traditional farms and were typical transhumance Fulani settlements that practice hand milking every morning. The milk from each cow (the four quarters) was pooled into a big jar that was meant for local consumption, production of cheese (locally called wara), or sold to milk off-takers. The breed of cattle was mostly white Fulani. The animals were extensively raised and occasionally supplemental feed and were provided when the farmers harvested their crops. Four pooled milk samples were collected at each of the 51 farms visited from lactating animals from dairy herders (farmers). Each sample (3–4 ml of milk and 50 g of cheese) were collected aseptically into a non-EDTA bottle, labeled and preserved in ice packed, and transported to the molecular laboratory of the Department of Veterinary Public Health and Preventive Medicine at the University of Ilorin for further processing.

### DNA extraction

For each milk sample, DNA was extracted using the DNeasy Blood and Tissue Kit (Qiagen, Milan, Italy) according to the manufacturer’s instructions. Before extraction, milk samples (500µL each) were prepared by centrifugation of whole milk at 1000 g for 10 min as previously described by Basanisi et al., [7]. The supernatant with milk fat layer was carefully removed, and the cell fraction was resuspended in 2 ml of phosphate-buffered saline (PBS). The re-suspended samples were centrifuged for 15 min at 1700 g and the supernatant was discarded. The resuspension of the pellet in PBS and centrifugation for 15 min at 1700 g was repeated until the residual cream was removed. The final sample preparation involved resuspension of the pellet which was resuspended and subjected to extraction in 180 µL of buffer ATL as described in the DNeasy Blood and Tissue Kit manufacturer’s instructions. For each cheese sample, 25 g was homogenized with a stomacher, and DNA isolation was performed on 25 g of the homogenate using the DNeasy Blood and Tissue Kit (Qiagen, Milan, Italy) following the manufacturer’s instructions.

### PCR assay conditions

The real-time PCR for the *Coxeilla burnetii IS1111* gene was performed using the Biobase LEIA-X4 instrument for genomic amplification using the following parameters: initial denaturation at 95^◦^C for 10 minutes followed by 45 cycles of 95^◦^C for 15 seconds and 58^◦^C for 1 minute. Each 20 µl probe-independent real-time PCR reaction mix consisted of 10µl of the Sso Advanced Universal SYBR Green Supermix (BioRad, Italy), 6µl of the *IS1111* primer (forward; 5’-CGGGTTAAGCGTGCTCAGTAT-3’, reverse; 5’-TCCACACGCTTCCATCACCAC-3’) [13], and 4 µl of purified DNA template. Data were analyzed with the Biobase real-time PCR software. We used double distilled water as a negative control throughout the reactions and used the DNA of an in-house *C. burnetii* isolate as the positive control. A cycle threshold (Ct) value of 36 or lower was used as the threshold for the analysis of isolates.

### True prevalence of *Coxiella burnetii*

We used the open-access – estimating true prevalence- tool provided by AUSVET (https://epitools.ausvet.com.au/trueprevalence) to determine the true prevalence of *C. burnetii* in milk samples in Ilorin, Kwara state. The tool requires the following parameters: the apparent prevalence of *C. burnetii*, the desired confidence interval (95% in this case), and the sensitivity and sensitivity of the diagnostic assay, as well as the desired confidence intervals for both the apparent and true prevalence estimations. The tool then used these parameters to determine the true prevalence and its 95% Blaker’s exact confidence interval.

## Results

The real-time PCR results indicated a notable presence of *C. burnetii*, the bacteria responsible for Q fever, in both milk and cheese samples from the sampled Fulani transhumance farms. The real-time PCR revealed that the *IS1111* gene was detected in 13 of the 69 isolates. Hence, the apparent prevalence of *C. burnetii* in the sampling sites was 18.8%. The results showed regional variation. There were significant differences in the burden of *C. burnetii* in milk between the three LGAs with a higher prevalence in Moro LGA (28.6%, *n* = 6/21) (Tables 1 and 2). In terms of product type, both dairy products showed similar prevalence with a relatively higher positivity rate in cheese (22.2%, *n* = 4/18) than in raw milk samples (17.6%, *n* = 9/51) (Table S1). The average CT value was 22.94 ± 3.72 within a range of 16.0 to 31.40.

**Table 1:** Test of association between the milk sampling location and the occurrence of *Coxiella burnetii* in milk and cheese

Sampling communities	Pooled milk samples (*n* = 51)				
	Positive n (%)	Negative n (%)	χ^2^	P-value	
Ilorin East (*n* = 9)	0 (0.0)	9 (100.0)	8.505	0.014	
Ifelodun (*n* = 27)	3 (11.1)	24 (88.9)			
Moro (*n* = 15)	6 (40.0)	9 (60.0)			
**Sampling communities**	**Cheese samples** (*n*** = 18**)				
	**Positive n (%)**	**Negative n (%)**	**χ** ^**2**^	**P-value**	
Ilorin East (*n* = 6)	1 (16.7)	5 (83.3)	5.345	0.069	
Ifelodun (*n* = 6)	3 (50.0)	3 (50.0)			
Moro (*n* = 6)	0 (0.0)	6 (100.0)			

With a diagnostic sensitivity of 81% and specificity of 90% [4], the true prevalence of *C. burnetii* was estimated to be 12.5% with a 95% Blacker Confidence limit of 1.9–27.6%. The positive predictive value was 0.54 whereas the negative predictive value was 0.97 (Table 2).

**Table 2:** Burden of *C. Burnetii* in dairy products in Kwara State, Nigeria (*n* = 69)

	Real-time PCR		Prevalence	
Sampling communities	Positive *n* (%)	Negative *n* (%)	Apparent (%)	True (95% confidence interval)
Ilorin East (*n* = 15)	1 (6.7)	14 (93.3)	6.7	12.5 (1.9–27.6)
Ifelodun (*n* = 33)	6 (18.2)	27 (81.8)	18.2	
Moro (*n* = 21)	6 (28.6)	15 (71.4)	28.6	

## Discussion

To our knowledge, this is the first study to utilize real-time PCR for the detection of *C. burnetii* in Nigeria. Our findings revealed the presence of this pathogen in milk and cheese, thus, a public health challenge. The prevalence reported by real-time PCR in milk (17.6%) was similar to the previous findings of our seroprevalence study (15.6%) that we conducted in Kwara State in 2020 [16]. In other parts of the country, several other studies reported prevalence rates that ranged from 14.5% of large ruminants being seropositive to *C. burnetii* in Kaduna State [35] to 13% seropositive in slaughtered cattle in Jalingo central abattoir, Taraba State [29]. In the Ibarapa rural livestock settlement in Oyo State, the seroprevalence of Q fever using iELISA was reported to be 23.5% [10]. In 2018, a study reported a significantly lower (6.2%) seroprevalence of *C. burnetii* in cattle in their cross-sectional seroprevalence study in cattle herds in Maigana and Birnin Gwari agro-ecological zone of Kaduna State, Nigeria [1]. In the core North, a study reported an adjusted *C. burnetii* herd-level seroprevalence of 40.36% (95%CI: 22.57–63.17%) [11]. Globally, significant variations in the prevalence of *C. burnetii* have been reported with a very low prevalence (3.57%) reported in milk samples in Brazil [14] and a prevalence of 16% was reported in bulk tank milk in Turkey [41]. A significantly higher prevalence rate was reported in bulk tank milk samples in the United States (94.3%, Kim et al., [22]. The positivity rate in cheese (22.2%) was similar to the pooled prevalence of 25.2% (95% confidence interval [CI]: 13.1–39.7%) reported by Yanmaz and Ozgen [42], Studies have reported the occurrence of *C. burnetii* in cheese to range from 5.6% in Turkey [40]; to 7.1 to 7.5 in Iran [20, 27]; and 85% in Poland [32]. These differences in prevalence could be attributed to different disease burdens across the sampling sites, the time (season or year) of sampling, and the differences in the analytical sensitivity of the tests.

Based on disease progression, real-time PCR could offer more diagnostic sensitivity and specificity than sero-surveillance using ELISA for many infectious diseases [18, 37]. In 2019, Bae et al. reported the diagnostic usefulness of molecular detection of *C. burnetii* from the blood of patients with suspected acute Q fever in a tertiary-care teaching hospital in Seoul, Republic of Korea. The study reported that *C. burnetii* PCR significantly improved early diagnosis of Q fever in patients as opposed to the use of serological testing which requires antibody formation approximately 2 to 3 weeks after onset of symptoms before they could be detected [4]. Another study reported that despite several animals showing high antibody titers for sustained periods, only one animal tested positive by PCR (Cq value of 38) for *C. burnetii* [39]. Also, it was reported that the analytical sensitivity of high throughput PCR to detect *C. burnetii* was excellent as a blind test on 183 clinical samples revealed a specificity of 100% (142/142) and the sensitivity was 71% (29/41) [18].

As an endemic and under-reported disease, the surveillance of *C. burnetii* could be improved using real-time PCR assay. The evidence of the exposure to *Coxiella* infection in ready-to-eat food products such as raw milk and cheese is worrisome because, aside from its negative effect on reproductive performance such as abortion, stillbirth, and reduced productivity of livestock, it is a persistent and highly transmissible pathogen to humans [15, 16]. In addition, the relatively higher prevalence in heat-processed and ready-to-eat cheese samples than in raw milk samples screened is an indication of a greater public health concern. *C. burnetii* bacteria are excreted in bodily fluids including milk and milk products, which serves as the longest-lasting source of human infection and cows are often asymptomatic but shed *C. burnetii* mainly in milk [15, 38].

With an apparent prevalence of 18.8% and an estimated true prevalence of 12.5%, this study showed that Q-fever is endemic in Kwara State in fresh dairy products screened. At a population level, we estimate that at least 100,000 adult cows are shedding the pathogen in their milk daily (based on an adult cattle population of 500,000–1,000,000 heads in the state (unpublished data from the Kwara State Ministry of Agriculture and Rural Development). This relatively high shedding could be attributed to the general lack of awareness of Q-fever among the pastoralists and dairy products retailers as well as the sharing of water and feeding fields during grazing and transhumance movements of the pastoralists [10].

Available evidence from literature on *C. burnetii* in Nigeria has shown significant variations among the several risk factors that were associated with the burden of *C. burnetii* in cattle which could equally affect the burden of the pathogen in ready-to-eat dairy products such as raw milk and cheese. For instance, factors such as the age of the cattle did not affect the seropositivity of cattle although there were no positive *C. burnetii* cases in calves less than one year of age [16]. The herd size was reported by Cadmus et al., [10] to be associated with seropositivity and could also affect the real-time PCR results as the higher the herd size, the more quantity of milk to be pooled and the more likely of real-time PCR detection of the pathogen upon sampling. While there was no statistically significant association between the different sampling points for our seroprevalence study in 2020, our current study showed significant variations between samples collected from each of the three sampling points with more cases in Ifelodun and Moro (six cases each) than in Ilorin East (Table 1). These differences could be attributed to differences in the sampling population, and the rural nature of the LGAs which would support larger livestock activities and thus larger herd sizes.

Milk is an ideal sample for *C. brunetii* screening as we earlier reported that the sex of animals was not a statistically significant risk factor for the seroprevalence of *C*. *burnetii* antibodies in both cattle and small ruminants sampled [16]. However, a past study from Nigeria found that the rate of infection was slightly higher among females than males and attributed this to the fact that the organism has a high affinity for the placenta, fetal membranes, and mammary glands and is found in large numbers in these tissues [35]. Most of the other risk factors that were studied to examine their association with seropositivity to *C. burnetii* such as the presence of ticks, the reproductive status of the animals, or their management style were more likely to impact the occurrence of the disease in animal hosts but not in milk samples.

Since infected ruminants can shed *C. burnetii* and other zoonotic pathogens in their milk, pasteurization of raw milk (and other milk products such as cheese) was introduced to ensure the inactivation of *C. burnetii* and other milk-borne pathogens mostly based on recommendations of the Codex Alimentarius [2, 12, 25]. It is therefore essential to educate farmers on the need for pasteurization at the appropriate temperature as well as post-pasteurization handling of milk products to reduce the burden of this disease. In addition, the various initiatives aimed at improving the dairy value chain in Nigeria must intensify screening for zoonotic milk-borne pathogens such as *C. burnetii*. Furthermore, other important endemic milk-borne zoonotic pathogens such as *Mycobacterium bovis* (zoonotic tuberculosis), *Brucella* sp. (brucellosis), *Salmonella* spp., *Listeria* spp., *E. coli*, *Campylobacter* spp., and *Clostridium* spp. could be transmitted through the consumption of unpasteurized milk [25, 26]. Also, animals seropositive to *C. burnetii* had 2.52 (95% CI: 2.29–2.77, *p*-value < 0.01) higher odds of being *Leptospira*-seropositive than those that were seronegative [39].

Generally, *C. burnetii* is a slow-growing bacterium that requires biosafety level 3 laboratories for microbial culture, Hence, a need for faster and more sensitive diagnostic tests such as ELISA, Complement fixation, or indirect immunofluorescence assay. While all sensitive, they appear only one to two weeks after infection [23]. Over the last two decades, newer diagnostic assays especially those based on PCR have been developed and validated to detect *C. burnetii* DNA in cell cultures and clinical samples. For instance, several studies have validated the clinical utility of conventional and nested PCR [23] and real-time PCR conditions [17, 23]. Also, studies have used the SYBR Green-based real-time PCR for early onset confirmation of *C. burnetii* and their antibiotic susceptibility profiles [9].

There are multiple copy numbers of a specific transposable element, the insertion sequence *IS1111*, in the genome of *C. burnetii* and are therefore routinely used for confirmation of Q fever cases (Duron, 2015). More recently, the *IS1111* gene has been used more frequently for the molecular confirmation of *C. burnetii* in milk samples [21, 22, 32, 39], The *IS1111*-based real-time PCR is very important due to its high diagnostic specificity and sensitivity and this was evidenced with a study that reported the presence of 14 cows shedding *C. burnetii* in milk without an appreciable serological response but positive by real-time PCR [6].

One of the main strengths of this study is its uniqueness and novelty in the study area. It is the first published work that utilized real-time PCR for the detection of *C. burnetii* in milk in Nigeria. Despite this, the following limitations may affect its widespread replication in Nigeria: Milk samples are usually pooled, so individual identification of shedding lactating cows is difficult. Also, adulteration of milk with water could affect the quality and concentration of extracted genomic materials which could affect the volume of DNA template needed per reaction. In addition, the presence of the *IS1111* gene in other Coxiella-like bacteria (endosymbionts) needs to be studied in milk to avoid misidentification with Coxiella-like endosymbionts.

## Conclusion

This study reiterates our earlier findings that dairy products are reservoirs of *C. burnetii* with a pooled prevalence of 18.8%. Its presence poses a direct risk of milk-borne zoonotic transmission of the disease to humans, particularly in Nigeria where the awareness about the disease is poor. We advocate that stakeholders involved in animal husbandry should be duly educated on the proper disposal of birth products as well as bodily fluids to reduce environmental contamination and aerosolization, persistence, and human infection. Secondly, public enlightenment campaigns against unpasteurized dairy products should be intensified. Thirdly, we propose a combined PCR and serology strategy for improving the routine and early diagnosis of Q fever in humans and animals. More molecular studies are needed to further study and characterize the *C. burnetii* genotypes in Nigeria.

## Electronic supplementary material

Below is the link to the electronic supplementary material.

Supplementary Material 1: Table S1. Real-time PCR detection of Coxiella burnetii from dairy samples in Ilorin, Kwara state, Nigeria.

## Data Availability

No datasets were generated or analysed during the current study.
