# Supplementary material for: Real-time PCR detection of Coxiella burnetii in dairy products in Kwara State, Nigeria: a public health concern
Source: BMC Vet Res. 2025 Jan 7;21:8. doi: 10.1186/s12917-024-04449-2 (PMC11706007; doi:10.1186/s12917-024-04449-2)
Supplement: Supplementary file 1 — Supplementary Material 1: Table S1. Real-time PCR detection of Coxiella burnetii from dairy samples in Ilorin, Kwara state, Nigeria. [file 12917_2024_4449_MOESM1_ESM.docx]

Table S1. Real-time PCR detection of *Coxiella burnetii* from dairy samples in Ilorin, Kwara state, Nigeria.

| **Sample type (ID)** | **Local Government Area** | **Ct Value** |
| --- | --- | --- |
| IDF-15 (Milk) | Ifelodun | 31.4 |
| ILR-EC1 (cheese) | Ilorin East | 22.5 |
| F0-001 (Milk) | Ifelodun | 18.6 |
| IFC-01 (cheese) | Ifelodun | 21.7 |
| IFC-2 (cheese) | Ifelodun | 20.1 |
| IFC-5 (cheese) | Ifelodun | 22.9 |
| IDF-22 (Milk) | Ifelodun | 16.0 |
| E02 (Milk) | Moro | 23.3 |
| E03 (Milk) | Moro | 26.3 |
| E04 (Milk) | Moro | 22.3 |
| E05 (Milk) | Moro | 24.2 |
| E06 (Milk) | Moro | 24.3 |
| E07 (Milk) | Moro | 24.6 |
